# Double staining method for array tomography using scanning electron microscopy

**DOI:** 10.1186/s42649-020-00033-8

**Published:** 2020-06-22

**Authors:** Eunjin Kim, Jiyoung Lee, Seulgi Noh, Ohkyung Kwon, Ji Young Mun

**Affiliations:** 1 National Instrumentation Center for Environmental Management, Seoul National University, Seoul, South Korea; 2 Department of Brain and Cognitive Sciences, Daegu Gyeongbuk Institute of Science & Technology (DGIST), Daegu, South Korea; 3 Neural circuit research group, Korea Brain Research Institute, Daegu, South Korea

**Keywords:** Array tomography, Double staining with uranyl acetate and lead, Scanning electron microscopy

## Abstract

Scanning electron microscopy (SEM) plays a central role in analyzing structures by imaging a large area of brain tissue at nanometer scales. A vast amount of data in the large area are required to study structural changes of cellular organelles in a specific cell, such as neurons, astrocytes, oligodendrocytes, and microglia among brain tissue, at sufficient resolution. Array tomography is a useful method for large-area imaging, and the osmium-thiocarbohydrazide-osmium (OTO) and ferrocyanide-reduced osmium methods are commonly used to enhance membrane contrast.

Because many samples prepared using the conventional technique without en bloc staining are considered inadequate for array tomography, we suggested an alternative technique using post-staining conventional samples and compared the advantages.

## Introduction

The brain has a densely connected neural network comprising several types of neurons and glial cells, including oligodendrocytes, microglia, and astrocytes. The study of how structures such as synapses and cellular organelles are regulated in a neural circuit requires techniques for imaging large areas at high resolution. Thin sections for transmission electron microscopy (TEM) are traditionally collected on fragile formvar film coated with one-hole TEM grids, but the area has a limitation. Preparing hundreds or thousands of serial thin sections of biological samples is complicated and only a few skilled technologists have mastered the technique (Hall et al. 2012). If the film breaks on one grid, serial imaging and volume reconstruction are unavailable. Scanning electron microscopy (SEM) provides new insights into large-area imaging through backscattered imaging and serial imaging compared TEM (Briggman and Bock 2012).

Serial imaging techniques during serial sectioning inside of the SEM chamber are common. Serial block-face scanning electron microscopy (SBEM) and focused ion beam SEM (FIB-SEM) are also popular techniques that use in situ destructive on-block sections inside the SEM vacuum chamber. DiK-SBEM uses a diamond knife (Denk and Horstmann 2004; Lippens et al. 2019), but FIB-SEM utilizes a focused ion beam (Bosch et al. 2015; Kubota et al. 2018b; Steyer et al. 2019). SBEM showed advances in volume EM in terms of reduced time for serial sectioning and alignment (Wanner et al. 2015). Array tomography and automatic tape ultramicrotomy (ATUM) (Baena et al. 2019) collect larger area serial thin sections than SBEM (Hayworth et al. 2014). In these techniques, samples exceeding the TEM grid size can be observed after placement of the sections on a SEM stub. The advantage of this technique is that the sections can be stored and large area imaging is available, compared to destructive sections of SBEM. SEM with MAPS or ATLAS 5, software for efficient navigation and auto-image acquisition using mosaics of adjacent images, can acquire large area images with a nanometer resolution (Hayworth et al. 2014).

For SEM imaging using SBEM and ATUM techniques, high contrast en bloc staining using reduced osmium-thiocarbohydrazide-osmium (rOTO), ferrocyanide-reduced osmium, uranyl acetate, and lead citrate are common (Hua et al. 2015). However, because heavy metals necessitate removing the charging effect during SEM imaging, several studies reported that these methods are challenging for immunostaining. Moreover, observing the specific contrast for post-synaptic density (de Vivo et al. 2017; Micheva et al. 2010; Oberti et al. 2011) is difficult. Therefore, we tested post-staining on large-area serial sectioning from conventional block without any en bloc staining and summarized the advantages of this alternative method.

## Main text

**Fig. 1:**
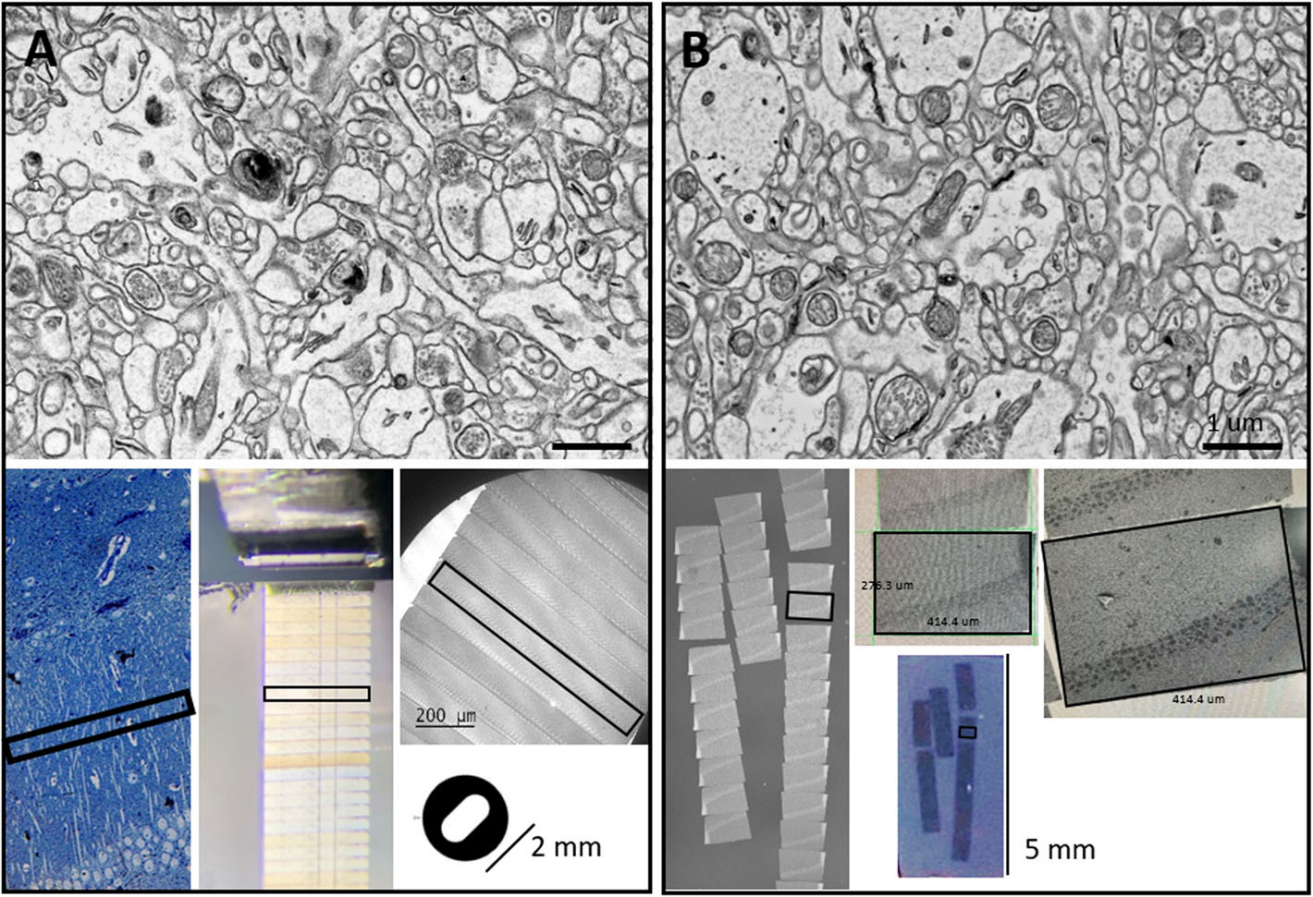
Comparison of TEM and SEM imaging. **a** TEM imaging. **b** SEM imaging. Both images were taken from thin sections of a block with osmium tetroxide, excess thiocarbohydrazide (TCH), and osmium tetroxide staining (OTO). For TEM imaging, the region of interest (100 * 800 μm) was trimmed, sectioned, and loaded onto a TEM grid. Unlike TEM, SEM imaging was selected from a large area (276.3 * 414.4 μm) in SEM without trimming before sectioning

Scanning electron microscopy (SEM)-based imaging uses the interaction of electrons with samples. The traditional SEM technique uses secondary electrons to investigate the surface characterization of samples. Conversely, back-scattered electrons are used for SEM imaging via interactions with heavy elements. Imaging using back-scattered electrons (Fig. 1b) is comparable to transmission electron microscopy (TEM; Fig. 1a). In both cases, good contrast is produced in brain tissue due to heavy metals with lipid components. Because thiocarbohydrazide (TCH) attaches to the osmium in brain tissue after osmification in fixation, more osmium binds to this site. The rOTO method increases membrane contrast in both TEM and SEM images (Fig. 1). In TEM, due to limitations of the TEM grid, serial sections are obtained 100 * 800 μm after trimming the interest area (Fig. 1a). Unlike TEM, SEM imaging can be used for large areas. In SEM, a 276.3 * 414.4 μm area can be sectioned serially, but the area of interest can be large. The study areas can be larger than 3 mm (depending on the knife size). A 4- or 8-in. wafer or 22 mm ITO glass is commonly used to load sections.

Three-dimensional electron microscopy (3DEM) is necessary for structural analysis of biological samples because a single cross-sectional image does not have sufficient information of complex 3D structures. Therefore, en bloc rOTO staining is commonly used for 3DEM, including SBEM and ATUM. However, rOTO has a few drawbacks. Because of the increase in membrane density, OTO sample sections can lose a relative amount of density in the mitochondrial matrix (Seligman et al. 1966), fine cellular membranes such as synaptic clefts, and post-synaptic area density (PSD) (Kubota et al. 2018a). A 30-nm wide synaptic cleft and expected dense contrast in the post-synaptic site was not shown in our images (Fig. 1). Because neurons transmit most trans-cellular signals through their synaptic contacts, neuroscientists analyze changing synaptic contacts depending on stimuli such as LTP, LDP, development, degeneration, and other factors. Therefore, decreasing the density of post-synaptic areas can be a disadvantage for neuroscientists. To reduce this disadvantage, Kubota et al. suggested mHMS staining based on rOTO protocol to detect synaptic contacts (Kubota et al. 2018a).

**Fig. 2:**
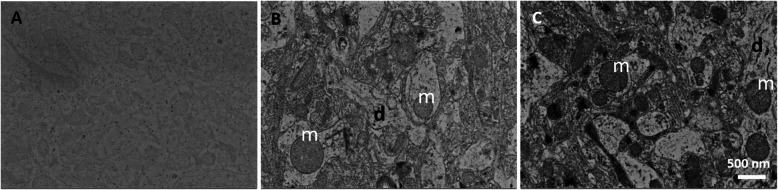
SEM imaging of one of section from a block without en bloc staining. SEM imaging of one section from a block without en bloc staining. **a** Without double staining. **b** Uranyl acetate in distilled water and lead citrate. **c** Uranyl acetate in 70% methanol and lead citrate. Double staining was effectively enhanced the contrast. Size bar = 500 nm, m: mitochondria, **d** dendrite

**Fig. 3:**
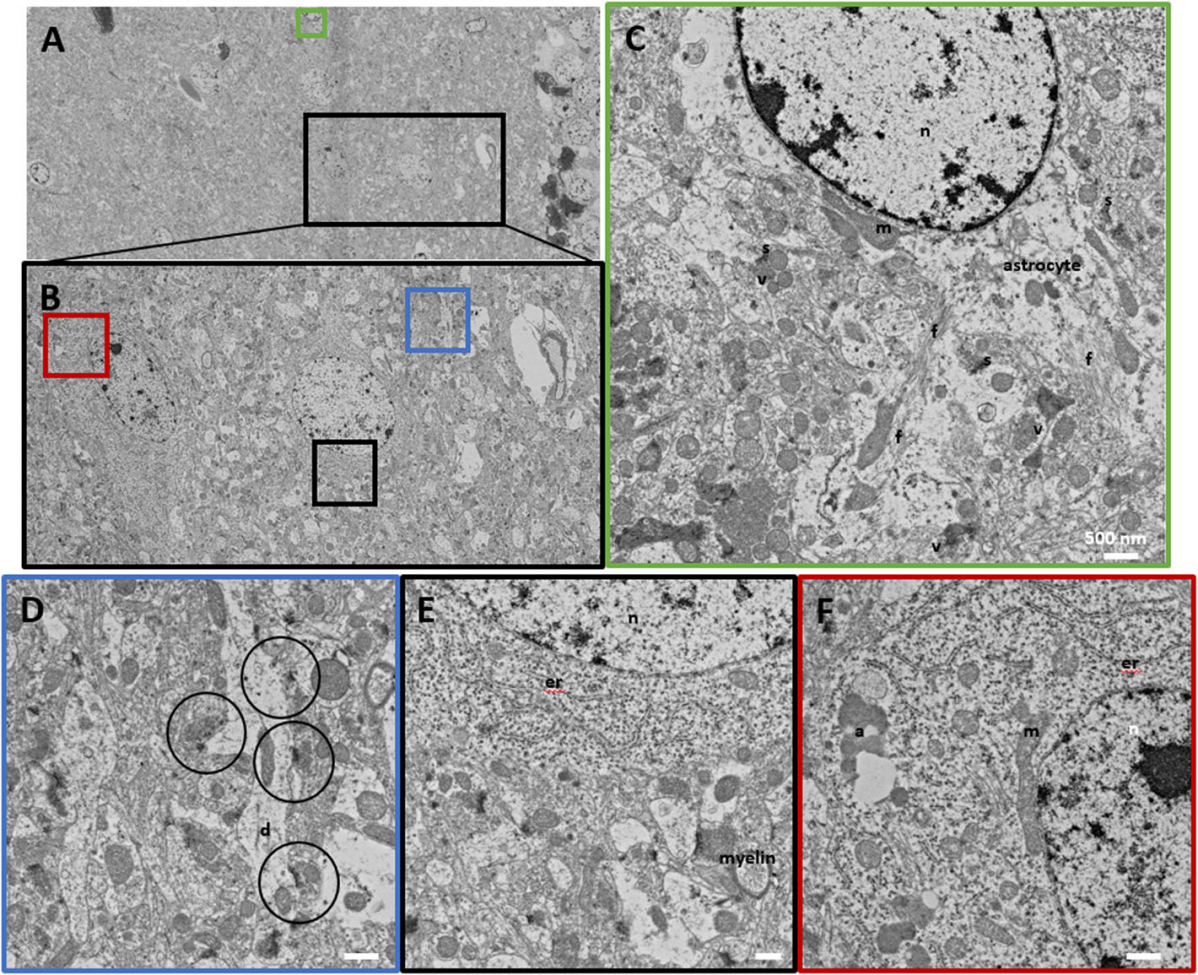
SEM imaging of a double-stained section. SEM imaging of a double-stained section. **a** 180 * 80 μm area, 5 nm/pixel image. **b** Black box in **a**, 60 μm * 35 μm. **c** Green box in **a**, 8 * 8 μm. **d** Blue box in **b**, 5.65 * 5.65 μm. **e** Black box in **b**, 5.65 * 5.65 μm. **f** Red box in **b**. Size bar = 500 nm; n: nucleus, m: mitochondria, d: dendrite, er: endoplasmic reticulum, a: autophagy, f: fibrils, v: synaptic vesicles, s: synapse, circle: dendritic spine

**Fig. 4:**
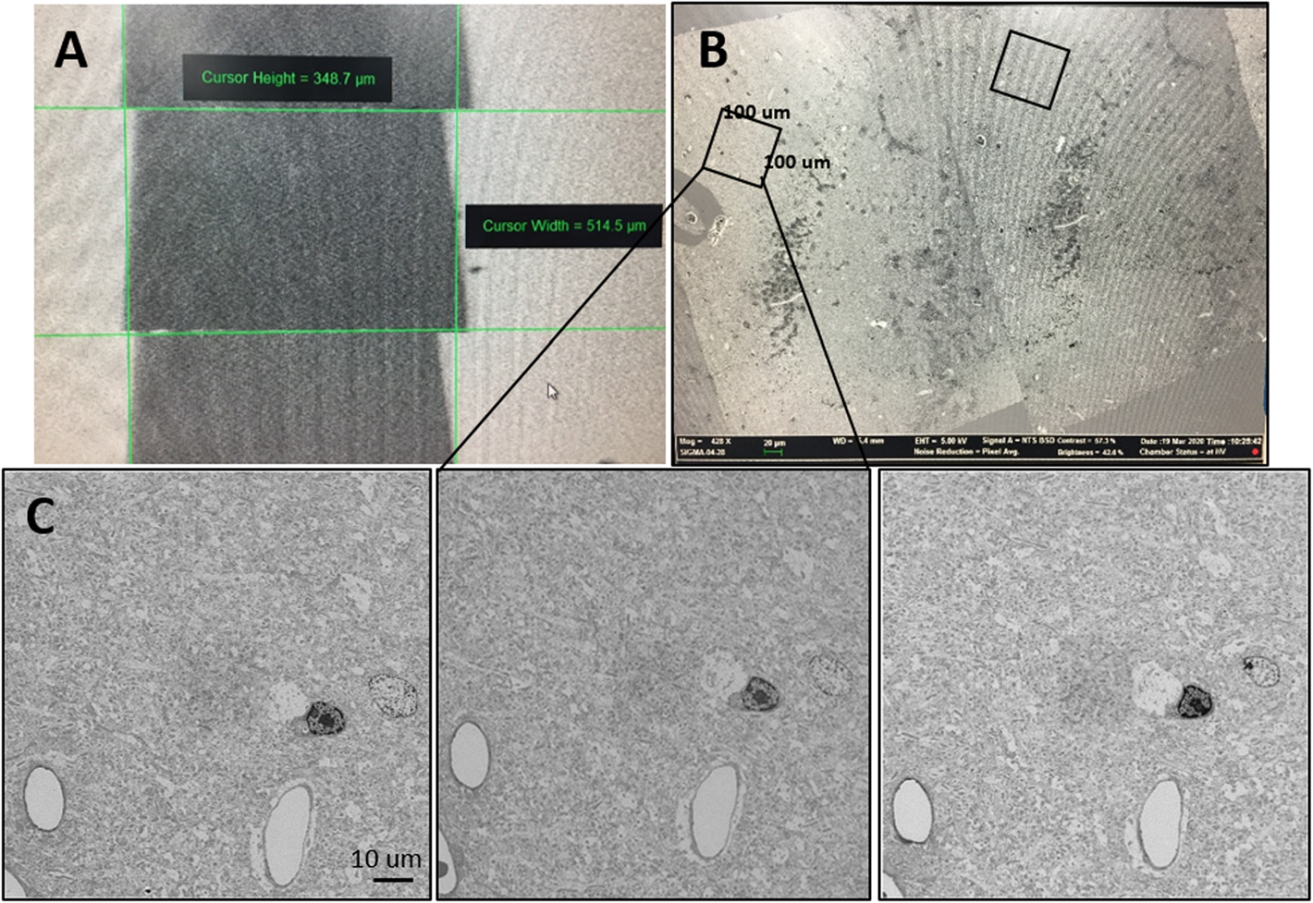
Serial imaging in SEM of a double-stained section. Serial imaging in SEM of a double-stained section. **a** 70 nm serial sectioning of a 348.7 * 514.4 μm area. **b** Selection of the SEM area of interest. **c** 3 Serial imaging of a 100 * 100 μm area using the ATLAS system

**Fig. 5:**
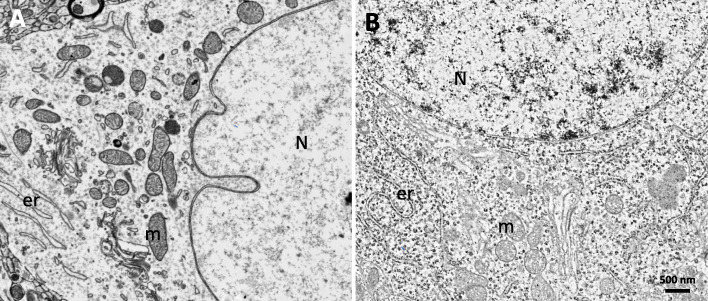
Direct comparison between rOTO sample and conventional sample with double staining in SEM. **a** 70 nm thickness section from rOTO sample. **b** 70 nm thickness section from conventional block with double staining. Both images showed good contrast to investigate cellular organelles such as mitochondria (m), nuclear (N), endoplasmic reticulum (er) in brain tissue

In this study, we suggest double staining with uranyl acetate and lead citrate after sectioning and loading a wafer from a block that does not have en bloc staining such as rOTO, uranyl acetate, and lead citrate. Figures 2, 3 and 4 shows that double staining with uranyl acetate and lead citrate on sections from a block without en bloc staining was effective to enhance their contrast (Figs. 2, 3 and 4). Figure 2 showed a comparison between no staining and double-stained sections. Without double staining, the section does not have sufficient contrast for SEM imaging (Fig. 2a), contrary to SBEM samples with rOTO en bloc staining (Fig. 1b). Uranyl acetate in methanol and lead staining (Fig. 2c) showed more contrast than uranyl acetate in distilled water and lead staining (Fig. 2b). In Fig. 3, several cell types were identified in 180 * 80 μm (Fig. 3a), and the selected area of interest (Fig. 3c-f) was analyzed. Figure 3c shows astrocytes in the hippocampus, which appear as star-shaped cells in light microscopy. Astrocytes are glial cells that are a pathological hallmark of brain responding lesions. In electron microscopy, numerous fibrils can be characterized as astrocyte features in this area (f in Fig. 3c). In TEM analysis, astrocytes are not easy to find because limitations in the TEM grid loading area. However, in large area SEM imaging, searching for a specific cell is easier than TEM imaging. Synaptic vesicles (v) and synapses (s) are clear in double-stained samples. Figure 3d shows clearer synapse and spines in the dendrites. The circles represent dendritic spines, synapses, and vesicles. Unlike SBEM sampling, the post-synaptic density (PSD) is clear with high contrast. Analyzing the synaptic strength has advantages. Cellular organelles such as the endoplasmic reticulum (er), autophagy (a), and mitochondria (m) were also observed in the cell body in Fig. 3e and f. For 3D reconstruction of a neural network, serial images were necessary. Figure 4 shows the workflow for SEM serial imaging. A 348.7 * 514.4 μm area (Fig. 4a) was observed at low magnification, and then the area of interest for specific neural circuits is selected under SEM (Fig. 4b). The 100 * 100 μm images were obtained by the ATLAS system for navigation. Three serial imaging images are shown in Fig. 4c. The images can be used for 3D reconstruction of synapse connections, cellular organelle communication, and networks between neurons and glial cells. For direct comparison, images from each blocks with or without rOTO staining were seen in Fig. 5. Sections from block without rOTO staining were observed after double staining. Both images showed sufficient contrast to observe cellular organelles in cell.

This technique is also very useful for correlative light and electron microscopy because too many heavy metals in en bloc-stained samples can mask specific antibody labeling antigens. Blocks without en bloc staining can be used for specific label immunostaining, and then double staining can improve their contrast for observing membranous structures.

## Materials and methods

The first sample preparation method followed SBEM sample preparation. Enhancing the signal is important for back-scattered electron imaging. To enhance the signal, brain tissue was treated with intensive osmium solution and en bloc heavy metal staining. These fixative and staining solutions helped obtain high membrane contrast and avoid severe charging (Hua et al. 2015; Wilke et al. 2013). Male mice (*n* = 2) were deeply anesthetized and intracardially perfused with 2% paraformaldehyde and 2.5% glutaraldehyde in 0.15 M cacodylate buffer (pH 7.4). Brain slices (150 μm thick) were produced with a vibratome in ice-cold 0.15 M cacodylate buffer, and small pieces of hippocampal CA1 region (SR) were incubated in the same fixative at 4 °C. After washing, the samples were placed in cacodylate buffer containing 4% OsO4/3% potassium ferrocyanide for 1 h. The tissues were placed in 1% thiocarbohydrazide (TCH) (Ted Pella, USA) solution for 20 min and then 2% aqueous OsO4 for 30 min. The tissues were then incubated in 1% uranyl acetate at overnight and lead aspartate solution for 30 min to enhance the membrane contrast as previously described. The tissues were dehydrated using a graded series of acetone (50%, 70%, 80%, 90%, 95%, and 100%) and infiltrated with a mixture of acetone and 100% resin. The resin was prepared using an Epon 812 kit (EMS, USA). The second sample followed conventional electron microscopy techniques without en bloc staining. The tissues were pre-fixed with 2.5% glutaraldehyde mixed with 2% paraformaldehyde solution (0.1 M phosphate buffer, pH 7.4) for 2 h, followed by post-fixation with 2% osmium tetroxide for 1 h. The samples were dehydrated with a graded ethanol series of 50%, 70%, 80%, 90%, 95%, 100%, and 100% and embedded in Spurr’s medium (Electron Microscopy Sciences, USA). Then, 70-nm-thick sections were produced using an ultramicrotome (Leica, UC7, Germany) and mounted on an TEM grid or silicon wafer. The sections from the second sample were double-stained with 2% uranyl acetate in D. W or 3% uranyl acetate in 70% methanol for 10 min and 0.2% lead citrate for 3 min (Table 1). If osmium pepper appeared, the sections were treated with 1% sodium periodate. The sections were viewed under a Tecnai G2 (FEI, USA) TEM at 200 kV, and an ATLAS 5 with an FESEM (Carl Zeiss, Germany) was used for mosaic imaging at 5 kV voltage and a 5 nm/pixel resolution.

**Table 1:** Double staining methods

Post-staining method	First staining	Second staining
UA in DW	2% uranyl acetate in DW	Lead citrate
UA in methanol	3% uranyl acetate in methanol	Lead citrate

## Conclusions

Double staining with uranyl acetate and lead citrate on array tomography from blocks without en bloc staining showed clear membrane structures in scanning electron microscopy. The post-synaptic structure and most cellular organelles of each cell type were clear in this technique. The best advantage of this alternative technique is to use conventional blocks without OTO staining and shorten time for sample preparation. Therefore, double staining array tomography sections can be an alternative scanning electron microscopy technique for large area imaging.

## Data Availability

The datasets used and/or analyzed during the current study are available from the corresponding author on reasonable request.

## Abbreviations

SEM: Scanning electron microscopy
OTO: Osmium-thiocarbohydrazide-osmium
TEM: Transmission electron microscopy
SBEM: Serial block-face scanning electron microscopy
FIB-SEM: Focused ion beam SEM
ATUM: Automatic tape ultramicrotomy
